# Supplementary material for: A Novel 13q12 Microdeletion Associated with Familial Syndromic Corneal Opacification
Source: Genes (Basel). 2023 May 1;14(5):1034. doi: 10.3390/genes14051034 (PMC10218699; doi:10.3390/genes14051034)
Supplement: Supplementary file 1 [file genes-14-01034-s001.zip › genes-2250013-supplementary.pdf]

Gene	Location	Variant	**gnomAD AF** [1]	**CADD** [2]	**Revel** [3]	Associated Phenotypes	Interpretation
LOXHD1	18:44,056,924-44,237,183	c.1763 G>C; p.G579A	0	25.2	0.822	**Recessive** Nonsyndromic Hearing Loss [4]; Dominant Late-Onset Fuch's [5]	VUS
SLC16A12	10:91,190,056-91,295,351	c.113 G>A; p.R38Q	$1.6E10^{-5}$	13.71	0.105	Dominant Juvenile and Age-Related Cataracts, Microcornea, and Renal Glucosuria [6, 7]	VUS

Table S1: Genetic variants segregating in all three affected family members, their locations (hg19), their gnomAD allele frequencies, CADD and REVEL scores, and associated phenotypes. VUS is variant of uncertain significance.

Gene	Location	Human LOF Phenotype
CRYL1	13:20,977,808-21,099,996	Hearing Loss [8, 9]
IFT88	13:21,141,296-21,265,583	Retinal Degeneration; NS CL/CP [10, 11]
IL17D	13:21,275,652-21,297,237	N/A
EEF1AKMT1	13:21,302,870-21,348,100	N/A
XPO4	13:21,351,468-21,476,913	Laryngomalacia, Developmental Delay [8, 12]
LATS2	13:21,547,175-21,635,725	Cardiac Defects, Cancer Development [12, 13]
SAP18	13:21,140,119-21,149,097	N/A
SKA3	13:21,727,734-21,750,691	N/A
MRP63	13:21,750,797-21,753,223	N/A
MIPEPP3	13:21,872,264-21,967,062	N/A
ZDHHC20	13:21,946,710-22,033,442	N/A
MICU2	13:21,946,710-22,033,442	Neurodevelopmental Disorder [14]

Table S2: Genes within the microdeletion, their location (hg19), and any suggested loss of function (LOF) human phenotypes.

GO Biological Process	Fold Enrichment	Raw P-Value	FDR
External Encapsulating Structure Organization	8.73	2.93E-15	1.53E-11
Extracellular Structure Organization	8.79	2.53E-15	1.99E-11
Extracellular Matrix Organization	8.82	2.35E-15	3.69E-11
Tube Development	3.49	4.09E-09	1.60E-05
Blood Vessel Development	4.44	1.01E-08	3.18E-05
Vasculature Development	4.26	2.09E-08	4.69E-05
Collagen Fibril Organization	15.44	2.03E-08	5.30E-05
Tube Morphogenesis	3.52	2.72E-07	4.74E-04
Collagen Metabolic Process	13.5	3.23E-07	5.06E-04
Blood Vessel Morphogenesis	4.4	2.71E-07	5.31E-04
Cellular Response to Amino Acid Stimulus	11.13	1.24E-06	1.49E-03
Angiogenesis	4.72	1.21E-06	1.58E-03
Circulatory System Development	2.94	1.17E-06	1.67E-03
Ossification	4.97	1.57E-06	1.75E-03
Eye Development	4.29	1.77E-06	1.85E-03
Visual System Development	4.25	2.01E-06	1.97E-03
Cellular Response to Acid Chemical	10.04	2.52E-06	1.97E-03
Tissue Development	2.27	2.29E-06	2.00E-03
Sensory System Development	4.18	2.43E-06	2.00E-03
Animal Organ Morphogenesis	2.77	2.19E-06	2.02E-03
Response to Wounding	3.92	2.73E-06	2.04E-03
Anatomical Structure Morphogenesis	2.07	3.32E-06	2.36E-03
Cellular Response to Transforming Growth Factor Beta Stimulus	6.68	4.40E-06	3.00E-03
Odontogenesis	7.35	6.48E-06	3.77E-03
Regulation of Collagen Metabolic Process	14.71	6.45E-06	3.89E-03
Eye Morphogenesis	6.43	6.06E-06	3.96E-03
Response to Transforming Growth Factor Beta	6.39	6.38E-06	4.00E-03
Wound Healing	4.26	8.50E-06	4.76E-03
Cell Adhesion	2.66	1.10E-05	5.95E-03
Positive Regulation of Collagen Biosynthetic Process	19.06	1.28E-05	6.68E-03
Positive Regulation of Collagen Metabolic Process	18.38	1.49E-05	7.32E-03
Negative Regulation of Developmental Process	2.68	1.48E-05	7.48E-03
Supramolecular Fiber Organization	3.19	2.04E-05	9.70E-03
Enzyme-Linked Receptor Protein Signaling Pathway	3.06	2.16E-05	9.98E-03
Response to Amino Acid	7.29	2.27E-05	1.02E-02
Camera-Type Eye Development	4.01	3.35E-05	1.46E-02
Regulation of Collagen Biosynthetic Process	13.91	4.99E-05	2.12E-02
Response to Acid Chemical	6.43	5.25E-05	2.16E-02
System Development	1.64	5.42E-05	2.18E-02
Anatomical Structure Formation Involved in Morphogenesis	2.52	5.72E-05	2.24E-02
Sensory Organ Development	3.01	7.22E-05	2.76E-02
Cellular Process	1.16	7.75E-05	2.89E-02
Response to Endogenous Stimulus	2.18	8.92E-05	3.25E-02
Regulation of Biomineralization	7	9.47E-05	3.37E-02
Direct Ossification	44.12	1.00E-04	3.41E-02
Regulation of Smooth Muscle Cell Proliferation	5.8	1.05E-04	3.41E-02
Intramembranous Ossification	44.12	1.00E-04	3.49E-02
Osteoblast Differentiation	5.8	1.05E-04	3.49E-02
Multicellular Organism Development	1.56	1.46E-04	4.68E-02

Table S3: All significant Gene Ontology (GO) processes [15] for upregulated genes with an adjusted p-value of less than or equal to 1e-10. FDR is false discovery rate.

Supplemental References

1. Chen, S.; Francioli, L. C.; Goodrich, J. K.; Collins, R. L.; Wang, Q.; Alföldi, J.; Watts, N. A.; Vittal, C.; Gauthier, L. D.; Poterba, T.; et al. A genome-wide mutational constraint map quantified from variation in 76,156 human genomes. bioRxiv 2022.03.20.485034 (2022). https://doi.org/10.1101/2022.03.20.485034. (accessed on 15 March 2023).
2. Kircher, M.; Witten, D.M.; Jain, P.; O'Roak, B.J.; Cooper, G.M.; Shendure, J. A general framework for estimating the relative pathogenicity of human genetic variants. Nat. Genet. 2014, 46, 310–315. https://doi.org/10.1038/ng.2892.
3. Ioannidis, N.M.; Rothstein, J.H.; Pejaver, V.; Middha, S.; McDonnell, S.K.; Baheti, S.; Musolf, A.; Li, Q.; Holzinger, E.; Karyadi, D.; et al. REVEL: An Ensemble Method for Predicting the Pathogenicity of Rare Missense Variants. Am. J. Hum. Genet. 2016, 99, 877–885. https://doi.org/10.1016/j.ajhg.2016.08.016.
4. Yu, S.; Chen, W.X.; Zhang, Y.F.; Chen, C.; Ni, Y.; Duan, B.; Wang, H.; Xu, Z.M. Recessive LOXHD1 variants cause a prelingual down-sloping hearing loss: Genotype-phenotype correlation and three additional children with novel variants. *Int. J. Pediatr. Otorhinolaryngol.* **2021**, *145*, 110715. https://doi.org/10.1016/j.ijporl.2021.110715.
5. Riazuddin, S.A.; Parker, D.S.; McGlumphy, E.J.; Oh, E.C.; Iliff, B.W.; Schmedt, T.; Jurkunas, U.; Schleif, R.; Katsanis, N.; Gottsch, J.D. Mutations in LOXHD1, a recessive-deafness locus, cause dominant late-onset Fuchs corneal dystrophy. *Am. J. Hum. Genet.* **2012**, *90*, 533–539. https://doi.org/10.1016/j.ajhg.2012.01.013.
6. Kloeckener-Gruissem, B.; Vandekerckhove, K.; Nurnberg, G.; Neidhardt, J.; Zeitz, C.; Nurnberg, P.; Schipper, I.; Berger, W. Mutation of solute carrier SLC16A12 associates with a syndrome combining juvenile cataract with microcornea and renal glucosuria. *Am. J. Hum. Genet.* **2008**, *82*, 772–779. https://doi.org/10.1016/j.ajhg.2007.12.013.
7. Zuercher, J.; Neidhardt, J.; Magyar, I.; Labs, S.; Moore, A.T.; Tanner, F.C.; Waseem, N.; Schorderet, D.F.; Munier, F.L.; Bhattacharya, S.; et al. Alterations of the 5'untranslated region of SLC16A12 lead to age-related cataract. *Investig. Ophthalmol. Vis. Sci.* **2010**, *51*, 3354–3361. https://doi.org/10.1167/iovs.10-5193.
8. Firth, H.V.; Richards, S.M.; Bevan, A.P.; Clayton, S.; Corpas, M.; Rajan, D.; Van Vooren, S.; Moreau, Y.; Pettett, R.M.; Carter, N.P. DECIPHER: Database of Chromosomal Imbalance and Phenotype in Humans using Ensembl Resources. *Am. J. Hum. Genet.* **2009**, *84*, 524–533. https://doi.org/10/1016/j.ajhg.2009.03.010. (accessed on 18 January 2023).
9. Hoefsloot, L.H.; Roux, A.F.; Bitner-Glindzicz, M. EMQN Best Practice guidelines for diagnostic testing of mutations causing non-syndromic hearing impairment at the DFNB1 locus. Eur. J. Hum. Genet. 2013, 21, 1325–1329. https://doi.org/10.1038/ejhg.2013.83.
10. Chekuri, A.; Guru, A.A.; Biswas, P.; Branham, K.; Borooah, S.; Soto-Hermida, A.; Hicks, M.; Khan, N.W.; Matsui, H.; Alapati, A.; et al. IFT88 mutations identified in individuals with non-syndromic recessive retinal degeneration result in abnormal ciliogenesis. *Hum. Genet.* **2018**, *137*, 447–458. https://doi.org/10.1007/s00439-018-1897-9.
11. Barba, A.; Urbina, C.; Maili, L.; Greives, M.R.; Blackwell, S.J.; Mulliken, J.B.; Chiquet, B.; Blanton, S.H.; Hecht, J.T.; Letra, A. Association of IFT88 gene variants with nonsyndromic cleft lip with or without cleft palate. *Birth Defects Res.* **2019**, *111*, 659–665. https://doi.org/10.1002/bdr2.1504.
12. Sobreira, N.; Schiettecatte, F.; Valle, D.; Hamosh, A. GeneMatcher: A matching tool for connecting investigators with an interest in the same gene. Hum. Mutat. 2015, 36, 928–930. https://doi.org/10.1002/humu.22844. (accessed on 18 January 2023).
13. Furth, N.; Aylon, Y. The LATS1 and LATS2 tumor suppressors: Beyond the Hippo pathway. *Cell Death Differ.* **2017**, *24*, 1488–1501. https://doi.org/10.1038/cdd.2017.99.
14. Shamseldin, H.E.; Alasmari, A.; Salih, M.A.; Samman, M.M.; Mian, S.A.; Alshidi, T.; Ibrahim, N.; Hashem, M.; Faqeih, E.; Al-Mohanna, F.; et al. A null mutation in MICU2 causes abnormal mitochondrial calcium homeostasis and a severe neurodevelopmental disorder. *Brain* **2017**, *140*, 2806–2813. https://doi.org/10.1093/brain/awx237.
15. Mi, H.; Muruganujan, A.; Ebert, D.; Huang, X.; Thomas, P.D. PANTHER version 14: More genomes, a new PANTHER GO-slim and improvements in enrichment analysis tools. *Nucleic Acids Res.* **2019**, *47*, D419–D426. https://doi.org/10.1093/nar/gky1038.
